# Supplementary material for: The Cryo-EM structures of two amphibian antimicrobial cross-β amyloid fibrils
Source: Nat Commun. 2022 Jul 27;13:4356. doi: 10.1038/s41467-022-32039-z (PMC9329304; doi:10.1038/s41467-022-32039-z)
Supplement: Supplementary file 1 — Supplementary Information [file 41467_2022_32039_MOESM1_ESM.pdf]

Supplementary Information

The Cryo-EM Structures of two Amphibian Antimicrobial Cross-β Amyloid Fibrils

Robert Bücker, Carolin Seuring, Cornelia Cazey, Katharina Veith, Maria García-Alai, Kay Grünewald, and Meytal Landau

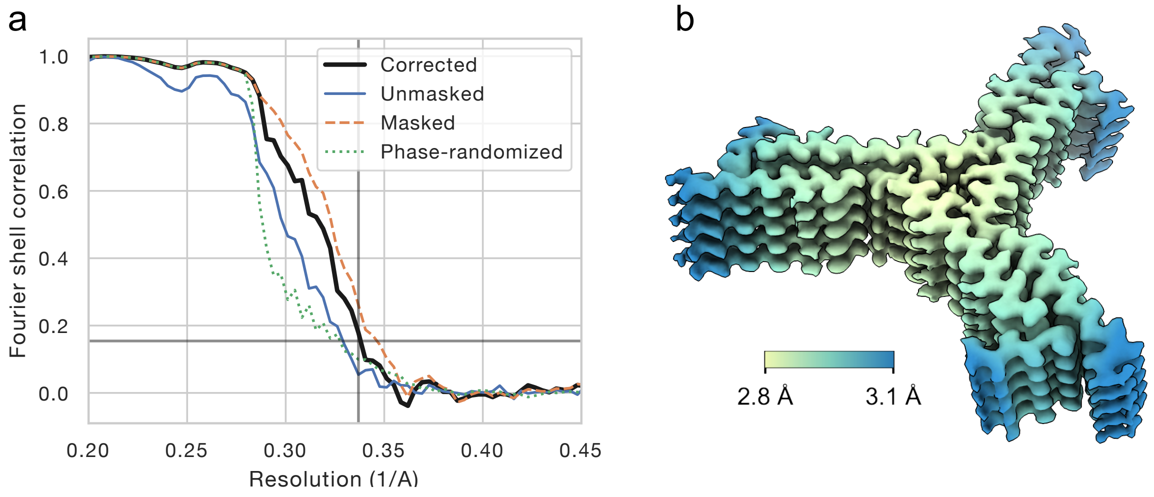

Supplementary Figure 1: (a) Half-set Fourier shell correlation for uperin 3.5 type I. The grey vertical line indicates the FSC=0.143 resolution of 3.0 Å, for a masked FSC after subtraction of a phase-randomized masked FSC (corrected, black line). (b) Local resolution map, calculated using half-set FSCs within a sliding window in Relion.

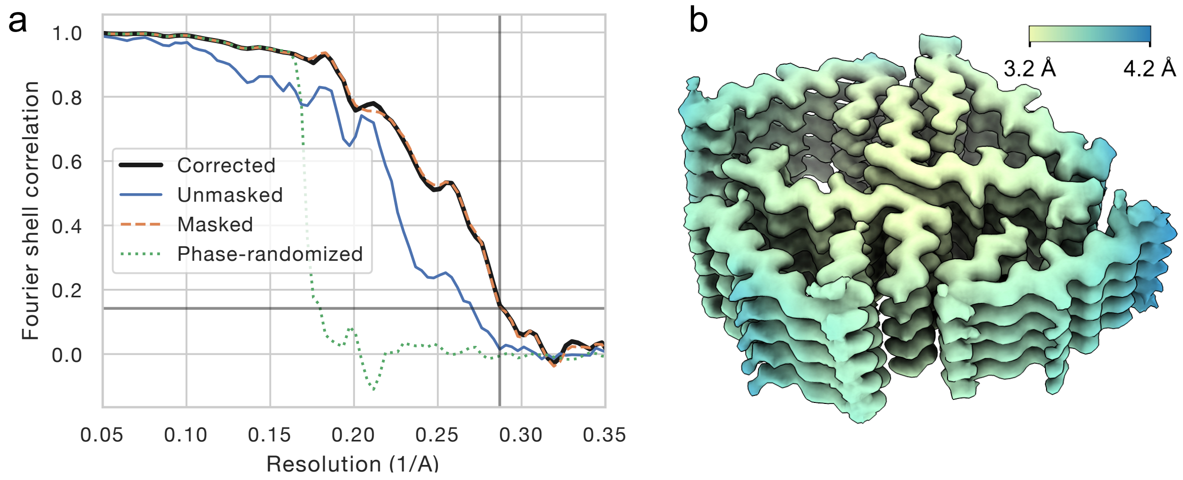

Supplementary Figure 2: (a) Half-set Fourier shell correlation for aurein 3.3. The grey vertical line indicates the FSC=0.143 resolution of 3.48 Å, for a masked FSC after subtraction of a phase-randomized masked FSC (corrected, black line). (b) Local resolution map, calculated using half-set FSCs within a sliding window in Relion.

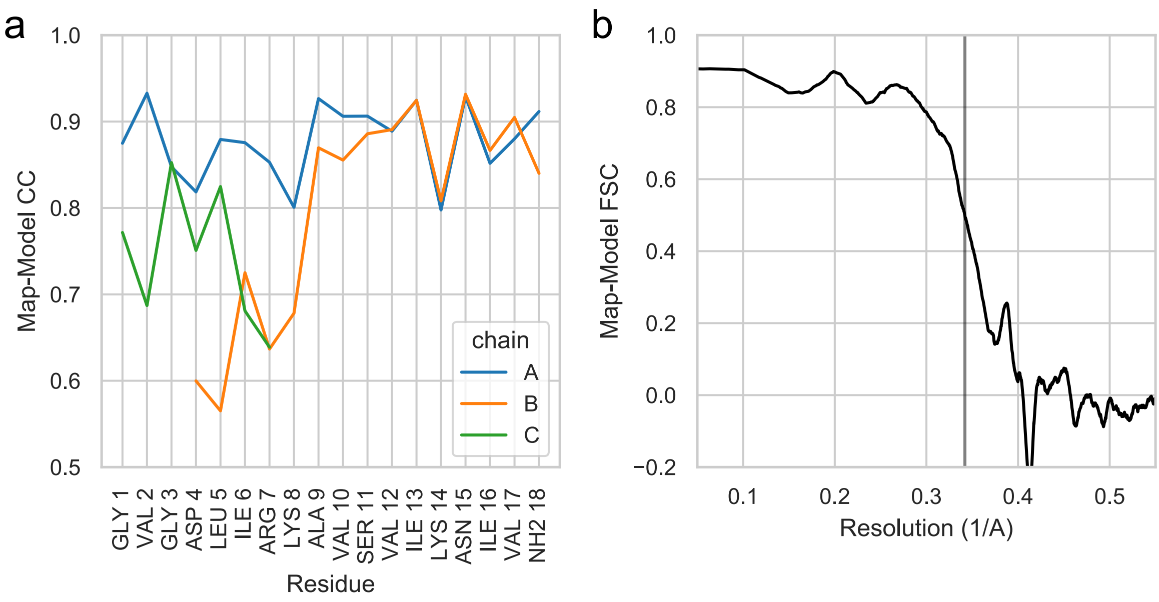

Supplementary Figure 3: (a) Map-model per-residue correlation coefficients computed at up to 3.0 Å and (b) map-model Fourier shell correlation for uperin 3.5. In (b), the vertical line indicates the FSC=0.5 value of 2.9 Å.

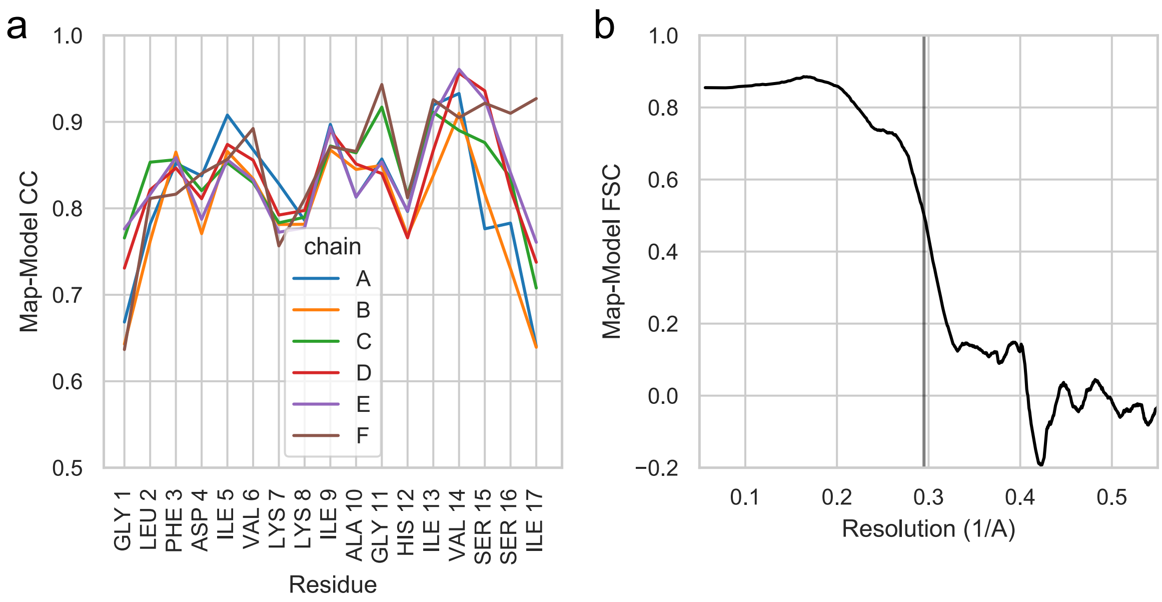

Supplementary Figure 4: (a) Map-model per-residue correlation coefficients computed at up to 3.5 Å and (b) map-model Fourier shell correlation for aurein 3.3. In (b), the vertical line indicates the FSC=0.5 value of 3.4 Å.

Supplementary Table 1: solvent-accessible surface area (SASA) buried within the uperin 3.5 and aurein 3.3 fibrils

**Uperin 3.5**	Chain A	Chain B	Chain C	Average
Chain SASA	2373Å^2	2040Å^2	1125Å^2	1846Å^2
Chain A	1422Å^2	231Å^2	147 Å^2	
Chain B	231Å^2	1255Å^2	136 Å^2	
Chain C	141Å^2	148Å^2	594 Å^2	
Chain A'	178Å^2	0Å^2	0Å^2	
Chain B'	82Å^2	0Å^2	0Å^2	
Chain C'	0Å^2	0Å^2	0Å^2	
Chain A''	177Å^2	79Å^2	0Å^2	
Chain B''	0Å^2	0Å^2	0Å^2	
Chain C''	0Å^2	0Å^2	0Å^2	
Axial SASA[a]				1091Å^2 (59%)
Lateral SASA[b]	745Å^2 (31%)	413Å^2 (20%)	266Å^2 (24%)	475Å^2 (26%)
Total SASA[c]	1723Å^2 (73%)	1399Å^2 (69%)	681Å^2 (61%)	1262Å^2 (69%)

**Aurein 3.3**	Chain A	Chain B	Chain C	Chain D	Chain E (inner)	Chain F (inner)	Average
Chain SASA	2326 Å^2	2299 Å^2	2304 Å^2	2302 Å^2	2321 Å^2	2317 Å^2	2312 Å^2
Chain A	1416 Å^2	0 Å^2	0 Å^2	0 Å^2	380 Å^2	0 Å^2	
Chain B	0 Å^2	1428 Å^2	0 Å^2	0 Å^2	114 Å^2	233 Å^2	
Chain C	0 Å^2	0 Å^2	1464 Å^2	32 Å^2	0 Å^2	394 Å^2	
Chain D	0 Å^2	0 Å^2	31 Å^2	1412 Å^2	78 Å^2	178 Å^2	
Chain E	394 Å^2	113 Å^2	0 Å^2	78 Å^2	1372 Å^2	121 Å^2	
Chain F	0 Å^2	235 Å^2	393 Å^2	176 Å^2	120 Å^2	1401 Å^2	
Axial SASA[a]							1416 Å^2 (61%)
Lateral SASA[b]	394 Å^2 (17%)	348 Å^2 (15%)	414 Å^2 (18%)	276 Å^2 (12%)	692 Å^2 (30%)	914 Å^2 (39%)	506 Å^2 (22%)
Total SASA[c]	1627 Å^2 (70%)	1608 Å^2 (70%)	1661 Å^2 (72%)	1542 Å^2 (67%)	1760 Å^2 (76%)	1881 Å^2 (79%)	1672 Å^2 (72%)

The solvent-accessible surface area (SASA) buried per chain was calculated at its interface with different sets of other chains as defined: a) The "Axial SASA" refers to the SASA buried by surrounding chains on the same sheet, indicated with the gray shading between all pairs. b) "Lateral SASA" refers to the SASA buried per chain by all other surrounding chains except the ones from the same β-sheet. Percentage of this buried area from the total SASA of the chain (first row) is indicated. a) "Total SASA" refers to the SASA buried per chain by all other surrounding chains in the fibril. The Percentage of this buried area from the total area of the chain (first row) is indicated.

Supplementary Table 2: Software and algorithms used

**Name**	**Reference**	**Purpose**
MotionCor2	https://emcore.ucsf.edu/ucsf-motioncor2/ (PMID:28250466)[1]	Motion correction of cryo-EM movies
crYOLO 1.8	https://cryolo.readthedocs.io/ (PMID:32627734)[2]	Automatic filament picking in micrographs
Relion 3.1	https://relion.readthedocs.io/ (PMID:32038040)[3]	Preprocessing, 2D and 3D classification, map auto-refinement, post-processing
CHEP 0.1.9	https://github.com/gschroe/chep (PMID: 33556421)[4]	Filament polymorph classification
cryoSPARC 2.3	https://cryosparc.com/ (PMID:28165473)[5]	Initial map building
Coot 0.93	https://www2.mrc-lmb.cam.ac.uk/personal/pemsley/coot/ (PMID:20383002)[6]	Structure model building
UCSF ChimeraX 1.3	https://www.rbvi.ucsf.edu/chimerax/ (PMID:28710774)[7]	Structure editing and visualization, model building
Isolde 1.0b3	https://isolde.cimr.cam.ac.uk/ (PMID:29872003)[8]	Structure model building/refinement
Phenix 1.19.1	https://www.phenix-online.org/ (PMID:20124702)[9]	Structure refinement and validation

1. Zheng, S. Q. *et al.* MotionCor2: anisotropic correction of beam-induced motion for improved cryo-electron microscopy. *Nat. Methods* **14**, 331–332 (2017).
2. Wagner, T. *et al.* Two particle-picking procedures for filamentous proteins: SPHIRE-crYOLO filament mode and SPHIRE-STRIPER. *Acta Crystallogr D Struct Biol* **76**, 613–620 (2020).
3. Scheres, S. H. W. Amyloid structure determination in RELION-3.1. *Acta Crystallogr D Struct Biol* **76**, 94–101 (2020).
4. Pothula, K. R., Geraets, J. A., Ferber, I. I. & Schröder, G. F. Clustering polymorphs of tau and IAPP fibrils with the CHEP algorithm. *Prog. Biophys. Mol. Biol.* **160**, 16–25 (2021).
5. Punjani, A., Rubinstein, J. L., Fleet, D. J. & Brubaker, M. A. cryoSPARC: algorithms for rapid unsupervised cryo-EM structure determination. *Nat. Methods* **14**, 290–296 (2017).
6. Emsley, P., Lohkamp, B., Scott, W. G. & Cowtan, K. Features and development of Coot. *Acta Crystallogr. D Biol. Crystallogr.* **66**, 486–501 (2010).
7. Goddard, T. D. *et al.* UCSF ChimeraX: Meeting modern challenges in visualization and analysis. *Protein Sci.* **27**, 14–25 (2018).
8. Croll, T. I. ISOLDE: a physically realistic environment for model building into low-resolution electron-density maps. *Acta Crystallogr D Struct Biol* **74**, 519–530 (2018).
9. Adams, P. D. *et al.* PHENIX: a comprehensive Python-based system for macromolecular structure solution. *Acta Crystallogr. D Biol. Crystallogr.* **66**, 213–221 (2010).
