# Supplementary material for: The Cryo-EM structures of two amphibian antimicrobial cross-β amyloid fibrils
Source: Nat Commun. 2022 Jul 27;13:4356. doi: 10.1038/s41467-022-32039-z (PMC9329304; doi:10.1038/s41467-022-32039-z)
Supplement: Supplementary file 2 — Reporting Summary [file 41467_2022_32039_MOESM2_ESM.pdf]

nature portfolio

Corresponding author(s): Meytal Landau

Last updated by author(s): 17/6/2022

Reporting Summary

Nature Portfolio wishes to improve the reproducibility of the work that we publish. This form provides structure for consistency and transparency in reporting. For further information on Nature Portfolio policies, see our Editorial Policies and the Editorial Policy Checklist.

Statistics

For all statistical analyses, confirm that the following items are present in the figure legend, table legend, main text, or Methods section.

n/a	Confirmed	
☐	☒	The exact sample size (*n*) for each experimental group/condition, given as a discrete number and unit of measurement
☐	☒	A statement on whether measurements were taken from distinct samples or whether the same sample was measured repeatedly
☒	☐	The statistical test(s) used AND whether they are one- or two-sided<br>*Only common tests should be described solely by name; describe more complex techniques in the Methods section.*
☐	☒	A description of all covariates tested
☐	☒	A description of any assumptions or corrections, such as tests of normality and adjustment for multiple comparisons
☒	☐	A full description of the statistical parameters including central tendency (e.g. means) or other basic estimates (e.g. regression coefficient) AND variation (e.g. standard deviation) or associated estimates of uncertainty (e.g. confidence intervals)
☒	☐	For null hypothesis testing, the test statistic (e.g. *F*, *t*, *r*) with confidence intervals, effect sizes, degrees of freedom and *P* value noted<br>*Give P values as exact values whenever suitable.*
☒	☐	For Bayesian analysis, information on the choice of priors and Markov chain Monte Carlo settings
☐	☒	For hierarchical and complex designs, identification of the appropriate level for tests and full reporting of outcomes
☒	☐	Estimates of effect sizes (e.g. Cohen's *d*, Pearson's *r*), indicating how they were calculated

Our web collection on statistics for biologists contains articles on many of the points above.

Software and code

Policy information about availability of computer code

Data collection	EPU 2.8.0.1256; Digital Micrograph 3.32.2403
Data analysis	CryoEM data processing: Relion 3.1.3, crYOLO 1.8-beta, MotionCor2, CHEP 0.1.9, cryoSPARC 3.2, Coot 0.9.3, ChimeraX 1.3, Phenix 1.19.1, Isolde 1.0b3

For manuscripts utilizing custom algorithms or software that are central to the research but not yet described in published literature, software must be made available to editors and reviewers. We strongly encourage code deposition in a community repository (e.g. GitHub). See the Nature Portfolio guidelines for submitting code & software for further information.

Data

Policy information about availability of data

All manuscripts must include a data availability statement. This statement should provide the following information, where applicable:

- Accession codes, unique identifiers, or web links for publicly available datasets
- A description of any restrictions on data availability
- For clinical datasets or third party data, please ensure that the statement adheres to our policy

The coordinates of the atomic models generated in this study have been deposited in the Protein Data Bank (PDB) under accession code 7QV5 [http://doi.org/10.2210/pdb7QV5/pdb] (uperin 3.5) and 7QV6 [http://doi.org/10.2210/pdb7QV6/pdb] (aurein 3.3). EM maps have been deposited at EMDB via the accession codes EMD-14167 [https://www.ebi.ac.uk/pdbe/entry/emdb/EMD-14167] (uperin 3.5 type I) and EMD-14168 [https://www.ebi.ac.uk/pdbe/entry/emdb/EMD-14168] (aurein 3.3).

Field-specific reporting

Please select the one below that is the best fit for your research. If you are not sure, read the appropriate sections before making your selection.

- [x] Life sciences
- [ ] Behavioural & social sciences
- [ ] Ecological, evolutionary & environmental sciences

For a reference copy of the document with all sections, see nature.com/documents/nr-reporting-summary-flat.pdf

Life sciences study design

All studies must disclose on these points even when the disclosure is negative.

Sample size	Uperin 3.5 Type I: 860080 unique helical units; Aurein 3.3: 141156 unique helical units
Data exclusions	Extracted image segments were filtered in 2D and 3D classification steps, following best practice in single-particle analysis
Replication	Hundreds of unique filaments were included into the analysis; for Uperin 3.5 the found variation (polymorphs) is discussed in the manuscript
Randomization	Not relevant for structure data processing
Blinding	Not relevant for structure data processing

Reporting for specific materials, systems and methods

We require information from authors about some types of materials, experimental systems and methods used in many studies. Here, indicate whether each material, system or method listed is relevant to your study. If you are not sure if a list item applies to your research, read the appropriate section before selecting a response.

Materials & experimental systems

n/a	Involved in the study	
x		Antibodies
x		Eukaryotic cell lines
x		Palaeontology and archaeology
x		Animals and other organisms
x		Human research participants
x		Clinical data
x		Dual use research of concern

Methods

n/a	Involved in the study	
x		ChIP-seq
x		Flow cytometry
x		MRI-based neuroimaging
